# The histone deacetylase inhibitor butyrate improves metabolism and reduces muscle atrophy during aging

**DOI:** 10.1111/acel.12387

**Published:** 2015-08-20

**Authors:** Michael E. Walsh, Arunabh Bhattacharya, Kavithalakshmi Sataranatarajan, Rizwan Qaisar, Lauren Sloane, Md M. Rahman, Michael Kinter, Holly Van Remmen

**Affiliations:** ^1^ Department of Cellular and Structural BiologySan AntonioTX78229; ^2^ The Barshop Institute for Longevity and Aging Studies, San Antonio, TX 78245The University of Texas Health Science Center at San AntonioTX78229USA; ^3^ Oklahoma Medical Research FoundationOklahoma CityOKUSA; ^4^ Department of BiologyState University of New York at DelhiDelhiNY15753USA

**Keywords:** sarcopenia, histone deacetylase, skeletal muscle, aging, metabolism, butyrate

## Abstract

Sarcopenia, the loss of skeletal muscle mass and function during aging, is a major contributor to disability and frailty in the elderly. Previous studies found a protective effect of reduced histone deacetylase activity in models of neurogenic muscle atrophy. Because loss of muscle mass during aging is associated with loss of motor neuron innervation, we investigated the potential for the histone deacetylase (HDAC) inhibitor butyrate to modulate age‐related muscle loss. Consistent with previous studies, we found significant loss of hindlimb muscle mass in 26‐month‐old C57Bl/6 female mice fed a control diet. Butyrate treatment starting at 16 months of age wholly or partially protected against muscle atrophy in hindlimb muscles. Butyrate increased muscle fiber cross‐sectional area and prevented intramuscular fat accumulation in the old mice. In addition to the protective effect on muscle mass, butyrate reduced fat mass and improved glucose metabolism in 26‐month‐old mice as determined by a glucose tolerance test. Furthermore, butyrate increased markers of mitochondrial biogenesis in skeletal muscle and whole‐body oxygen consumption without affecting activity. The increase in mass in butyrate‐treated mice was not due to reduced ubiquitin‐mediated proteasomal degradation. However, butyrate reduced markers of oxidative stress and apoptosis and altered antioxidant enzyme activity. Our data is the first to show a beneficial effect of butyrate on muscle mass during aging and suggests HDACs contribute to age‐related muscle atrophy and may be effective targets for intervention in sarcopenia and age‐related metabolic disease.

## Introduction

Loss of muscle mass and function during aging (sarcopenia) is characterized by molecular, histological and functional alterations in skeletal muscle. Sarcopenia contributes to reduced muscle strength and metabolic disease during aging and is associated with the loss of healthspan during aging. The causes of sarcopenia are not totally understood, but may include intrinsic factors like hormonal imbalance, inflammation, denervation, oxidative stress, mitochondrial dysfunction, and impaired satellite cell function and extrinsic factors like reduced physical activity and poor nutrition (e.g., low protein intake). The complexity of the potential modulators of sarcopenia underscores the fact that no pharmacological interventions exist to prevent the progressive deterioration in muscle mass and function during aging, although several interventions, including myostatin and hormonal therapies, are under investigation. Resistance exercise is the only intervention that is able to increase protein synthesis (Hasten *et al*., 2000) and strength in older adults, although this effect is blunted in adults over 80 years (Slivka *et al*., 2008).

In this study, we tested the effectiveness of a histone deacetylase inhibitor, butyrate, as an intervention to modulate sarcopenia. Protein acetylation affects a variety of cellular processes and is regulated by two classes of enzymes: histone acetyltransferases and histone deacetylases (HDACs). Histone acetyltransferases catalyze the addition of acetyl groups to lysine residues, and histone deacetylases remove acetyl groups from lysine residues. Although histones are the classic target of histone acetyltransferases and HDACs, thousands of proteins are acetylated in tissue‐specific manner, including the majority of skeletal muscle contractile proteins (Lundby *et al*., 2012) as well as most metabolic enzymes (Zhao *et al*., 2010).

Histone deacetylases play a critical role in skeletal muscle during development and adulthood. The class II HDACs regulate myogenesis via the transcription factor myocyte enhancer factor‐2 (Lu *et al*., 2000), and HDAC4 is involved in innervation‐regulated gene transcription (Tang & Goldman, 2006). HDAC4 inhibits Dach2, a negative regulator of myogenin, a transcription factor that regulates denervation‐induced gene expression. Mice deficient in HDAC4 and HDAC5 are protected against muscle loss in a surgical model of denervation; these mice do not upregulate the muscle‐specific E3 ligases *MuRF1* or *atrogin‐1* in response to denervation (Moresi *et al*., 2010). Furthermore, HDAC1, a class I HDAC, induces muscle atrophy in response to disuse and nutrient deprivation (Beharry *et al*., 2014). Thus, histone deacetylases are an attractive target for diseases that result in muscle atrophy.

In fact, pharmacological inhibitors of HDACs have also shown promise in preclinical models of diseases of muscle, including amyotrophic lateral sclerosis (Yoo & Ko, 2011), spinal muscular atrophy (Minamiyama *et al*., 2004) and muscular dystrophy (Consalvi *et al*., 2013). For example, HDAC inhibitors reduce fibrosis and improve muscle function in a mouse model of muscular dystrophy (Consalvi *et al*., 2013). Because pharmacological inhibitors of class I and II HDACs have been shown to ameliorate neuromuscular disease in animal models, we asked whether the general HDAC inhibitor butyrate could attenuate muscle atrophy and improve metabolism during aging.

Butyrate is a four‐carbon fatty acid that is produced in the gut as a product of fermentation. Butyrate is a general HDAC inhibitor and protects against high‐fat diet‐induced metabolic changes (Gao *et al*., 2009), has anti‐inflammatory properties (Maslowski *et al*., 2009) and extends lifespan in a mouse model of progeria (Krishnan *et al*., 2011) and *Drosophila* (Zhao *et al*., 2005). Thus, we hypothesized that butyrate would improve metabolism and prevent muscle atrophy in mice during aging. To test this, we fed adult mice a control diet and butyrate‐ containing diet and measured the effect on skeletal muscle mass, biochemical and physiological properties in aged mice.

## Results

### Butyrate increases histone acetylation in peripheral tissues and improves glucose metabolism during aging

**Figure 1:**
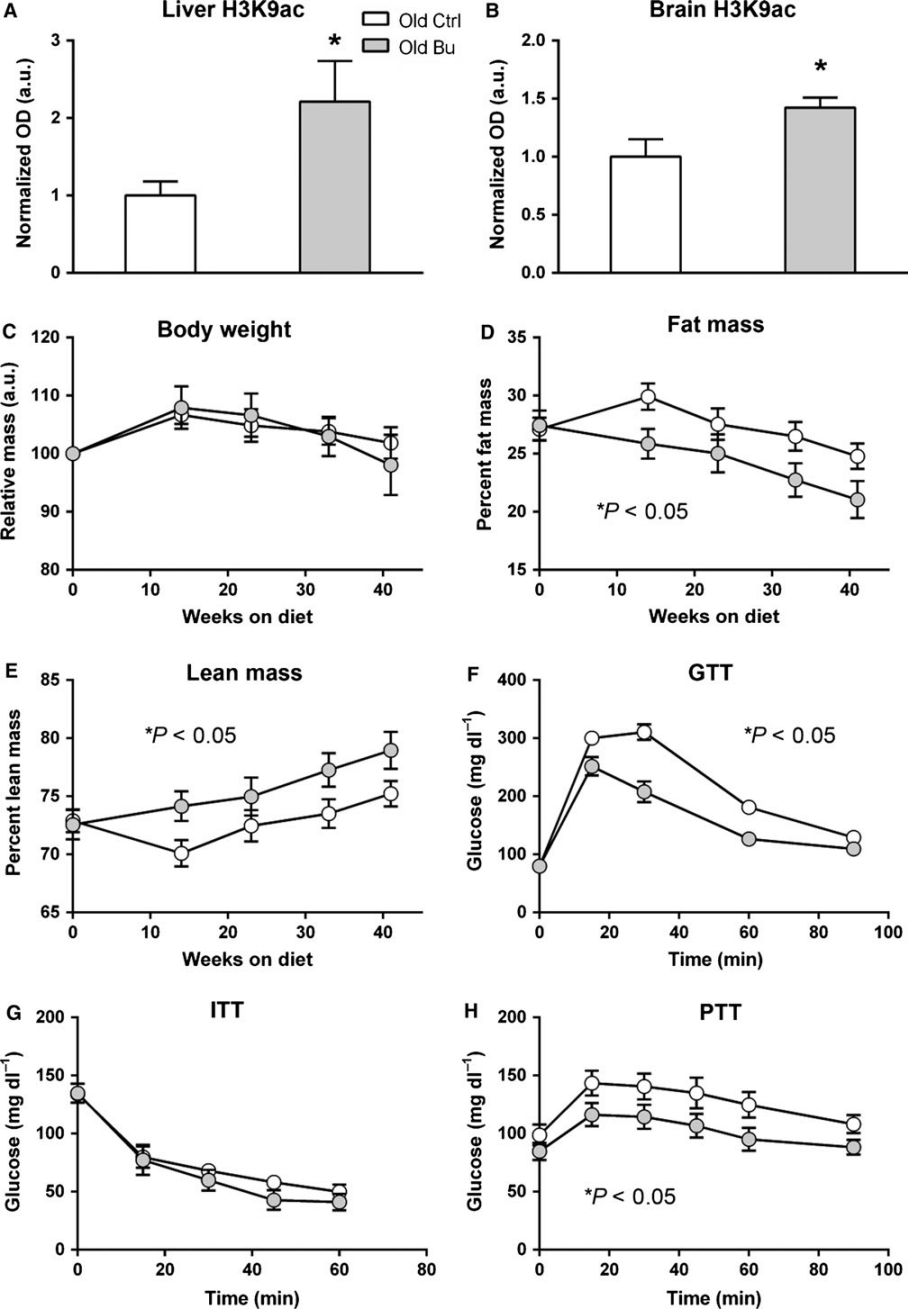
Butyrate reduces fat mass and improves glucose metabolism in C57Bl/6 mice during aging. Butyrate increases histone acetylation in (A) brain and (B) liver (*P < 0.05 vs. control). (C) Body weight changes over the 10‐month study. (D) Percent fat mass and (E) percent lean mass measured by quantitative magnetic resonance imaging in old mice (*n* = 14–22). Intraperitoneal (F) glucose, (G) insulin, and (H) pyruvate tolerance tests in old mice (*n* = 14–15 for GTT and PTT;* n* = 7 for ITT; control aged – white; butyrate aged – gray. *P < 0.05 analyzed by ANOVA with repeated measures).

Butyrate is a general histone deacetylase inhibitor; thus, we expected to find increased histone acetylation upon butyrate treatment. Consistent with a previous report that diet‐incorporated butyrate inhibits HDAC activity in peripheral tissues, including skeletal muscle (Gao *et al*., 2009), we found elevated histone acetylation at histone H3 lysine 9 in liver and brain (Fig. 1A,B) as determined by Western blot. To determine the effect of butyrate during aging, we fed 16‐month‐old female C57Bl/6 mice control or butyrate diets until 26 months of age and monitored body weight and body composition over the course of the study using quantitative magnetic resonance imaging. Butyrate did not affect body mass or composition in young mice (data not shown). In old mice, butyrate did not affect the change in body weight over the course of the study (Fig. 1C), but reduced percent fat mass (Fig. 1D), resulting in a concomitant increase in percent lean mass (Fig. 1E). The loss of fat mass was not due to changes in food consumption or body temperature (Fig. S1A,B, Supporting information).

Reduced fat mass is associated with improved glucose metabolism, and butyrate has been shown to prevent fat accumulation and insulin intolerance during high‐fat feeding (Gao *et al*., 2009). Thus, we tested the effect of butyrate on glucose, insulin and pyruvate tolerance in old mice. Butyrate improved glucose tolerance in old mice based on intraperitoneal glucose tolerance tests (Fig. 1F). However, butyrate had no effect during insulin tolerance tests in old mice (Fig. 1G), but improved pyruvate tolerance in old mice (Fig. 1H). Butyrate had no effect on glucose or pyruvate tolerance in young mice (not shown).

### Butyrate prevents hindlimb muscle loss during aging

**Figure 2:**
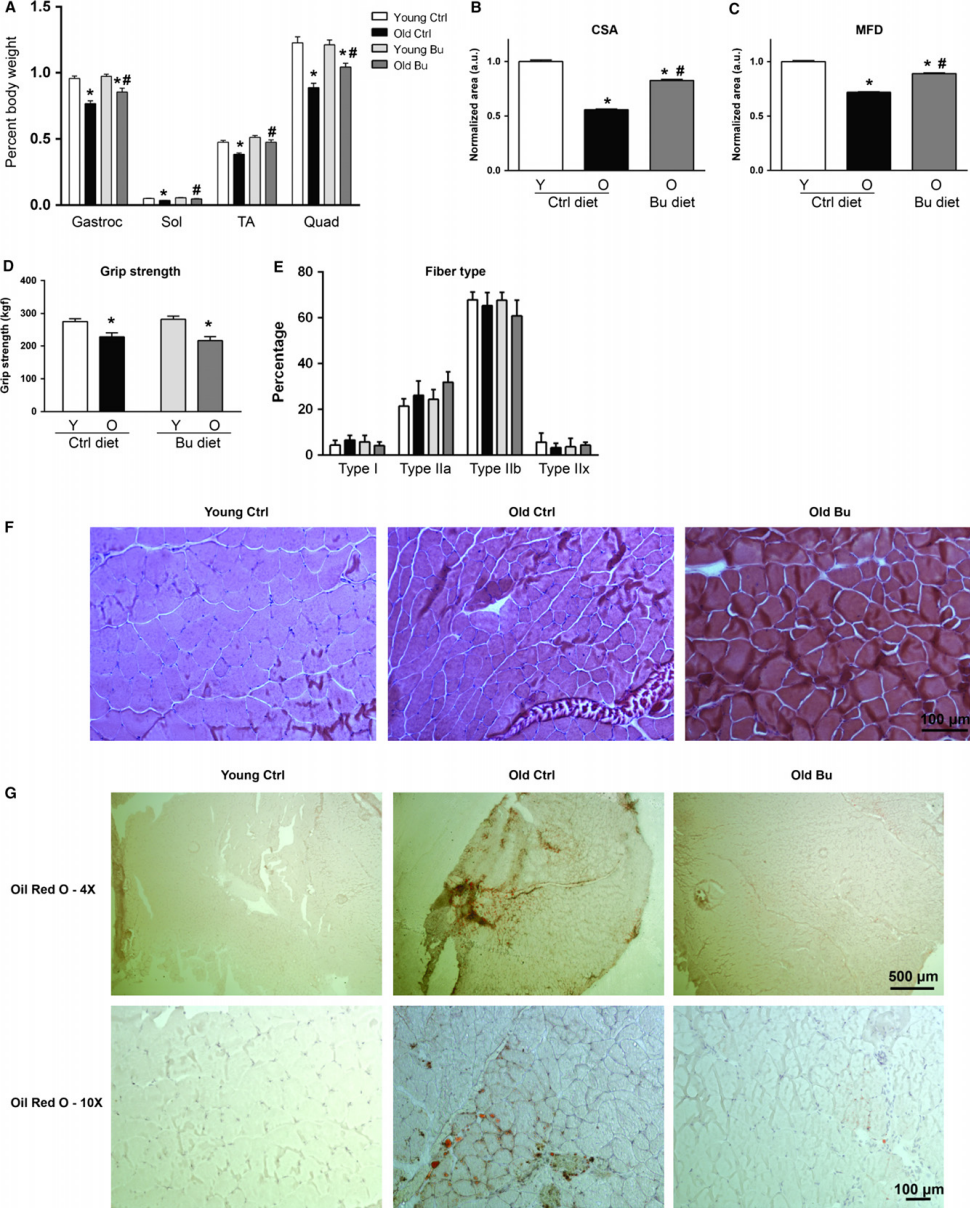
Butyrate prevents muscle atrophy during aging. (A) Hindlimb muscle mass of 12 and 26‐month‐old C57Bl/6 female mice relative to body weight (*n* = 14–17). (B) Cross‐sectional area and (C) minimum Feret's diameter (*n* = 3) from muscle sections stained with hematoxylin and treosin. (D) Four‐paw grip strength (*n* = 5–8). (E) Muscle fiber type determined by MHC isoform composition (*n* = 3–4). (F) Representative images used to quantify CSA and MFD (*P < 0.05 vs. diet‐matched young; ^#^ *P* < 0.05 vs. age‐matched control; control young – white, control aged – black, butyrate young – light gray, butyrate aged – dark gray). (G) Oil Red O staining of lipid droplets in gastrocnemius muscle (Sections from three animals were examined per group; 4× and 10× images are from two different animals per group). All data are displayed as means with standard error.

To determine whether the increase in lean mass in butyrate‐fed mice was due to preserved muscle mass, we measured the mass of a number of organs that contribute to lean mass. With the exception of skeletal muscle, butyrate had no effect on lean tissue weight during aging (Fig. S1C), although aging independently affected gonadal fat, liver, heart, and kidney mass. In the gastrocnemius–plantaris muscles, mice fed a control diet lost 20% of mass at 26 months of age, while butyrate‐treated mice lost only 11% (Fig. 2A), and in the tibialis anterior, control‐fed mice lost 19% of mass and butyrate‐treated mice lost 7% (*P* < 0.05). Similar results were found with the quadriceps and soleus muscles (Fig. 2A). In total, 26‐month‐old mice lost 23% of hindlimb muscle mass compared with diet‐matched 14‐month‐old mice, while butyrate treatment resulted in a loss of 12%. Although there was no effect of age or diet on body weight at sacrifice, hindlimb muscle wet weight revealed significant increases in mass of only the TA and soleus (Fig. S1E). While butyrate prevented muscle atrophy during aging, it had no effect on the age‐related decline in four‐paw grip strength (Fig. 2D). We estimated fiber‐type composition by measuring Type I and II myosin heavy chain (MHC) with SDS‐PAGE, and neither butyrate nor aging had an effect on the levels of MHC isoforms (Fig. 2E).

To determine whether butyrate prevented myofiber atrophy in old mice, we stained gastrocnemius–plantaris cross sections with hematoxylin and treosin (Fig. 2F) and measured myofiber cross‐sectional area (CSA) and minimum Feret's diameter (MFD), which removes potential error from the sectioning process (Briguet *et al*., 2004). Aging resulted in a 44% loss of CSA and a 28% loss in MFD in mice fed a control diet relative to young mice fed a control diet (Fig. 2B,C, *P* < 0.05), while mice fed butyrate had a reduction of 17% and 11% for CSA and MFD, respectively, relative to young mice fed a control diet. Muscle from young mice fed butyrate was not available for histology. Because intramuscular fat is known to be associated with metabolic disease, we stained gastrocnemius muscle for lipid accumulation. Aging resulted in the accumulation of large lipid droplets (Fig. 2G), while butyrate prevented this accumulation.

We also tested whether aging and butyrate had effects on other functional measurements, including bone mineral content and density, peripheral nerve function and memory, and found no significant effect of butyrate on any parameter (Fig. S2, Supporting information).

### Butyrate elevates markers of mitochondrial biogenesis in skeletal muscle

**Figure 3:**
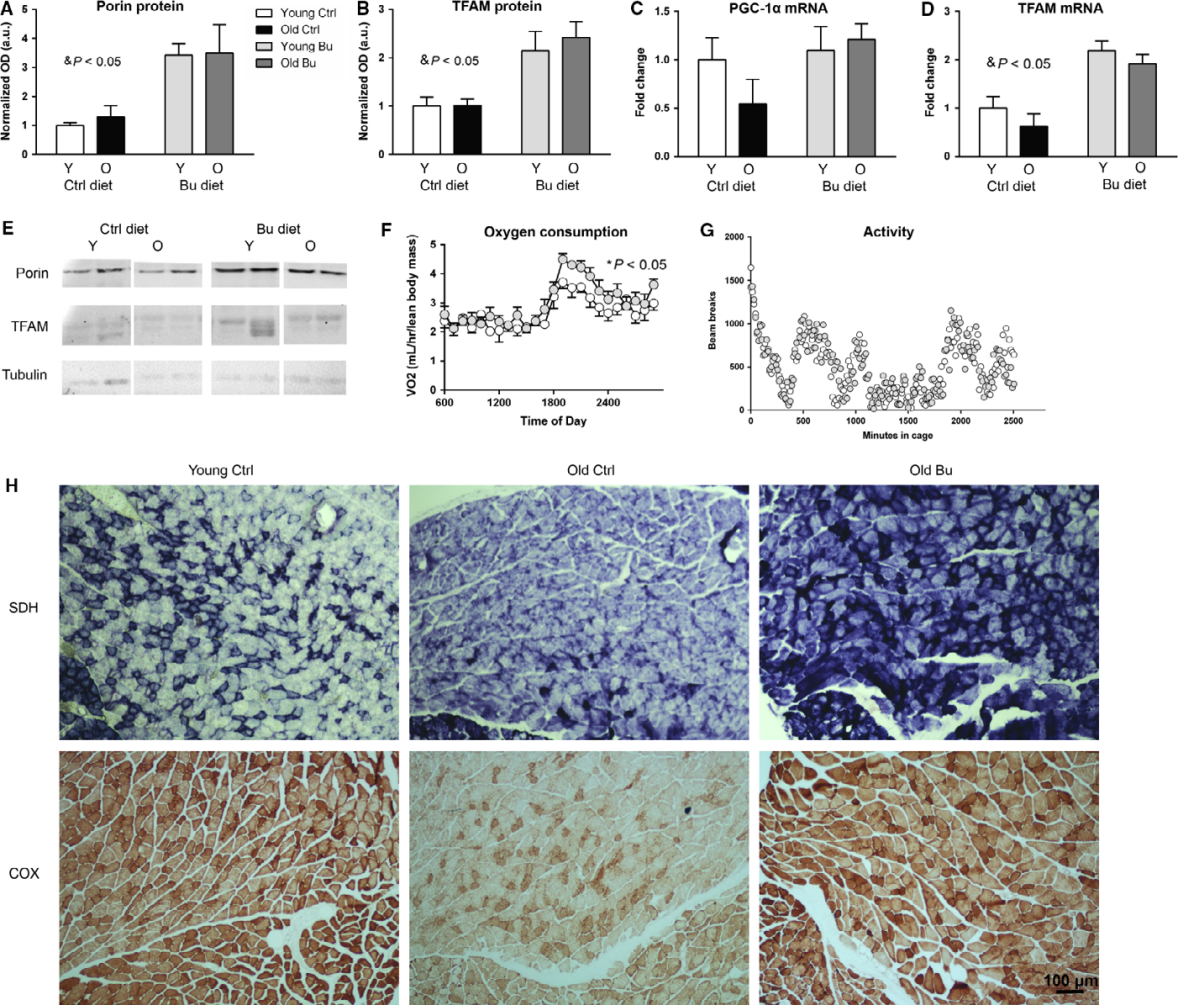
Butyrate increases mitochondrial biogenesis in skeletal muscle. Protein levels of (A) porin and (B) TFAM during aging and butyrate treatment were determined by Western blot. mRNA levels of (C) PGC‐1α and (D) TFAM in young and old animals treated with butyrate were determined by qPCR. (E) Western blot quantified in (A) and (B) (^&^ *P* < 0.05 significant effect of diet; control young – white, control aged – black, butyrate young – light gray, butyrate aged – dark gray). (F) Oxygen consumption over 24 h; dark cycle only is statistically significant (*n* = 8). (G) 40‐h spontaneous activity in old mice (*n* = 3–4) (control aged – white; butyrate aged – gray). (H) Top – Succinate dehydrogenase activity in gastrocnemius muscle from young control, old control, and old butyrate groups. Soleus muscle in the bottom left corner of each image. Bottom – Cytochrome C activity in gastrocnemius muscle with soleus muscle in the bottom of each image (Sections from three animals were examined per group).

**Figure 4:**
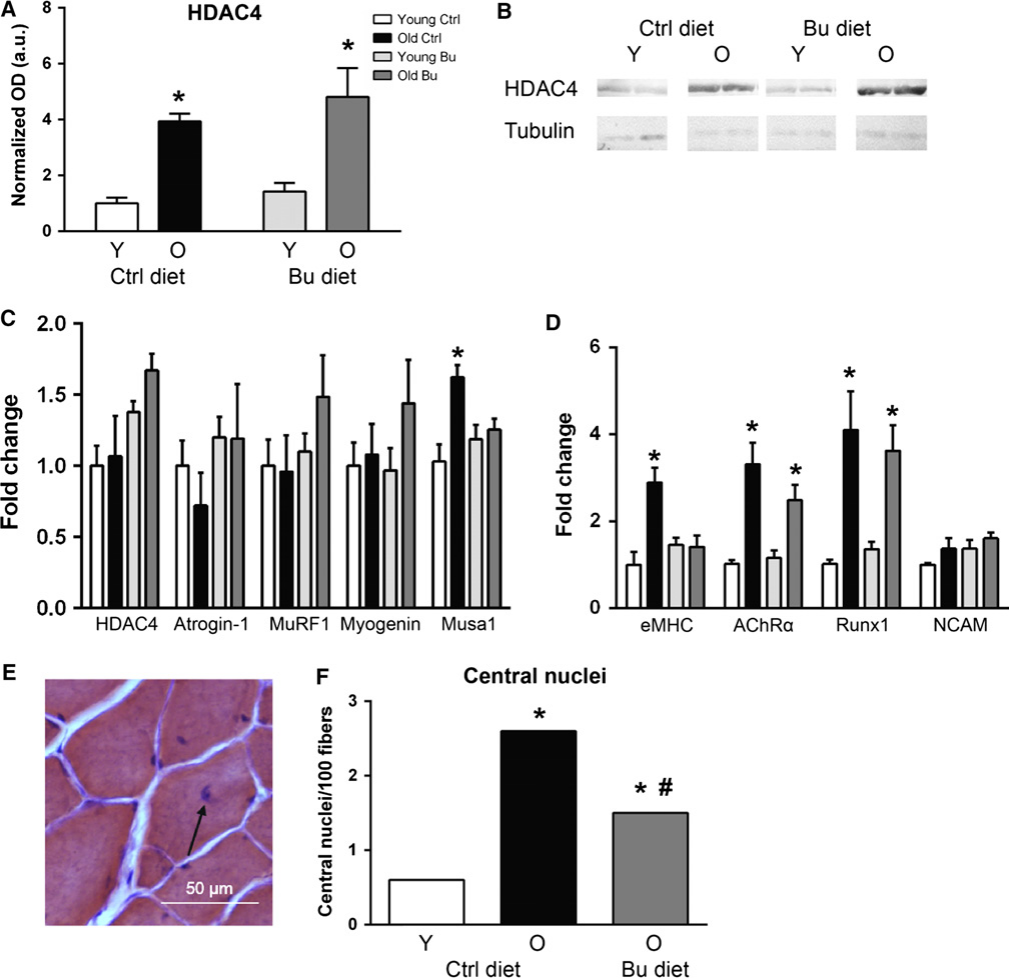
Effect of butyrate and aging on HDAC4/myogenin signaling, oxidative stress, and apoptotic markers. (A) HDAC4 protein was determined by (B) Western blot (*n* = 3). (C) Expression of HDAC4, myogenin, atrogin‐1, MuRF1, and Musa1 was determined by qPCR (*n* = 8). (D) Expression of embryonic MHC (eMHC), AChRα, Runx1, and neural cell adhesion molecule (NCAM) was determined by qPCR (*n* = 8; *P < 0.05 vs. diet‐matched young determined by ANOVA with Tukey's post‐test; data are displayed as means with standard error). (E) Central nuclei in a muscle fiber stained with hematoxylin and treosin (arrow). (F) Frequency of central nuclei per 100 muscle fibers (chi‐squared test was used to determine significance; *P < 0.05 vs. diet‐matched young; ^#^ *P* < 0.05 vs. age‐matched control; control young – white, control aged – black, butyrate young – light gray, butyrate aged – dark gray).

Mitochondrial function has been implicated in age‐related muscle loss, and previous findings suggested that butyrate elevates mitochondrial biogenesis in skeletal muscle (Gao *et al*., 2009). As shown in Figs 3A,B and 4E, butyrate increased levels of the mitochondrial proteins porin, an outer mitochondrial membrane associated protein, and mitochondrial transcription factor A (TFAM), a transcription factor for the mitochondrial genome. We also determined the levels of PGC1α and TFAM mRNA by qPCR. Neither aging nor butyrate affected PGC1α expression (Fig. 3C), and butyrate significantly increased TFAM levels (Fig. 3D).

Because mitochondrial biogenesis is associated with increased metabolic rate, we measured oxygen consumption using indirect calorimetry. Indeed, butyrate treatment increased oxygen consumption in the dark cycle, but not the light cycle, in old mice (Fig. 3F). Butyrate treatment did not affect respiratory quotient or resting metabolic rate in either the dark or light cycle in old mice (not shown). Oxygen consumption was not determined in young mice. The increase in oxygen consumption in old mice treated with butyrate was not due to changes in activity (Fig. 3G).

### Butyrate modulates oxidative, but not glycolytic, metabolic enzymes

We used a quantitative mass spectrometry approach to determine the effect of aging and butyrate on metabolic proteins in skeletal muscle. Using selected reaction monitoring, we quantified a comprehensive list of proteins involved in glycolysis in young and old animals treated with butyrate or control diets. Significant effects of age were detected in 11/19 proteins, including creatine kinase, lactate dehydrogenase b and phosphoglycerate kinase 1 (Table 1). Butyrate did not affect any glycolytic enzymes. We also determined the effect of butyrate and aging on a comprehensive list of proteins involved in oxidative metabolism. A significant effect of age was detected in 5/25 proteins (Table 1). Further, butyrate significantly affected 3/25 proteins, including isocitrate dehydrogenase 2 and succinate dehydrogenase (SDH), and an age–diet interaction was detected in two proteins (fumarate hydratase 1 and isocitrate dehydrogenase 3a).

**Table 1:** Effect of age and butyrate on enzymes involved in glycolytic and oxidative metabolism

	Young Ctrl	Old Ctrl	Young Bu	Old Bu	Significance
Glycolytic metabolism (pmol per 100 μg total protein)					
AldoA	22.0 ± 2.0	19.6 ± 1.4	22.0 ± 2.0	17.5 ± 1.0	
Ckm	72.9 ± 3.1	33.8 ± 10.9	59.0 ± 10.3	46.3 ± 4.5	*
Ckmt2	2.9 ± 0.32	2.0 ± 0.76	2.8 ± 0.56	3.3 ± 0.78	
Eno1	0.45 ± 0.03	0.57 ± 0.07	0.52 ± 0.01	0.54 ± 0.04	
Eno3	19.6 ± 1.5	17.9 ± 0.85	19.9 ± 2.2	15.1 ± 1.2	
Gapdh	8.3 ± 1.3	6.2 ± 1.4	9.1 ± 2.0	4.5 ± 0.89	*
Gpi1	0.80 ± 0.04	0.56 ± 0.10	0.75 ± 0.03	0.64 ± 0.05	*
Hk1	0.04 ± 0.003	0.03 ± 0.002	0.04 ± 0.004	0.03 ± 0.002	*
Ldha	16.7 ± 0.88	17.2 ± 1.2	18.0 ± 1.4	15.4 ± 1.4	
Ldhb	0.21 ± 0.03	0.34 ± 0.05	0.26 ± 0.02	0.47 ± 0.07	*
Pc	0.06 ± 0.01	0.03 ± 0.005	0.05 ± 0.01	0.03 ± 0.004	*
Pfkm	0.72 ± 0.11	0.36 ± 0.12	0.71 ± 0.20	0.28 ± 0.07	*
Pgam2	12.4 ± 0.66	14.5 ± 1.3	13.0 ± 1.1	10.8 ± 0.71	
Pgk1	2.6 ± 0.14	1.5 ± 0.41	2.5 ± 0.34	2.0 ± 0.14	*
Pkm2	8.2 ± 0.41	6.6 ± 0.83	8.6 ± 0.47	6.6 ± 0.32	*
Pygm	2.1 ± 0.35	0.90 ± 0.33	1.7 ± 0.51	0.75 ± 0.22	*
Slc2a4	0.28 ± 0.01	0.19 ± 0.05	0.28 ± 0.05	0.24 ± 0.01	
Tkt	0.06 ± 0.005	0.12 ± 0.021	0.05 ± 0.005	0.09 ± 0.005	*
Tpi1	5.0 ± 0.39	3.6 ± 0.49	5.2 ± 0.47	3.8 ± 0.26	
Oxidative metabolism (pmol per 100 μg total protein)					
Aco2	1.2 ± 0.12	0.90 ± 0.24	1.3 ± 0.22	1.6 ± 0.24	
Cs	1.78 ± 0.14	1.64 ± 0.35	1.89 ± 0.15	2.26 ± 0.27	
Dlat	0.53 ± 0.05	0.48 ± 0.12	0.60 ± 0.08	0.67 ± 0.10	
Dld	0.17 ± 0.01	0.22 ± 0.01	0.22 ± 0.02	0.25 ± 0.02	&
Dlst	0.64 ± 0.08	0.51 ± 0.07	0.76 ± 0.08	0.66 ± 0.09	
Etfdh	0.10 ± 0.01	0.07 ± 0.02	0.08 ± 0.01	0.14 ± 0.03	
Fh1	0.39 ± 0.03	0.27 ± 0.06	0.38 ± 0.06	0.45 ± 0.08	^
Idh1	0.05 ± 0.003	0.03 ± 0.002	0.04 ± 0.003	0.02 ± 0.002	*
Idh2	0.32 ± 0.05	0.29 ± 0.09	0.31 ± 0.07	0.80 ± 0.12	*, &
Idh3a	0.38 ± 0.04	0.32 ± 0.07	0.43 ± 0.06	0.40 ± 0.05	^
Idh3b	0.06 ± 0.01	0.03 ± 0.01	0.06 ± 0.02	0.04 ± 0.01	
Idh3g	0.36 ± 0.07	0.22 ± 0.08	0.35 ± 0.06	0.31 ± 0.09	
Mdh1	2.22 ± 0.18	2.36 ± 0.29	2.62 ± 0.23	3.24 ± 0.47	
Mdh2	2.60 ± 0.20	3.11 ± 0.39	2.93 ± 0.30	3.74 ± 0.54	
Ogdh 2	0.55 ± 0.06	0.36 ± 0.08	0.55 ± 0.09	0.63 ± 0.08	
Pdha1	0.56 ± 0.05	0.41 ± 0.11	0.61 ± 0.08	0.68 ± 0.10	
Pdhb	0.27 ± 0.02	0.20 ± 0.05	0.29 ± 0.03	0.34 ± 0.04	
Pdk2	0.06 ± 0.004	0.05 ± 0.013	0.06 ± 0.010	0.06 ± 0.011	
Pdp1	0.08 ± 0.010	0.02 ± 0.004	0.09 ± 0.013	0.02 ± 0.003	*
Sdha	0.17 ± 0.02	0.17 ± 0.03	0.20 ± 0.02	0.28 ± 0.03	&
Sdhb	0.18 ± 0.02	0.18 ± 0.04	0.21 ± 0.03	0.27 ± 0.04	
Sdhc	0.03 ± 0.004	0.02 ± 0.005	0.04 ± 0.007	0.02 ± 0.003	*
Slc25a11	0.11 ± 0.023	0.02 ± 0.009	0.10 ± 0.028	0.02 ± 0.007	*
Sucla2	0.11 ± 0.02	0.06 ± 0.02	0.12 ± 0.03	0.11 ± 0.02	
Suclg1	0.11 ± 0.004	0.10 ± 0.021	0.08 ± 0.013	0.12 ± 0.014	

Glycolytic enzyme levels were determined by quantitative mass spec in skeletal muscle (*n* = 6; pmol per 100 μg total protein; data shown are means with standard deviation.

*Significant effect of age; ^&^Significant effect of diet; ^^^Significant effect of age*diet interaction).

**Figure 5:**
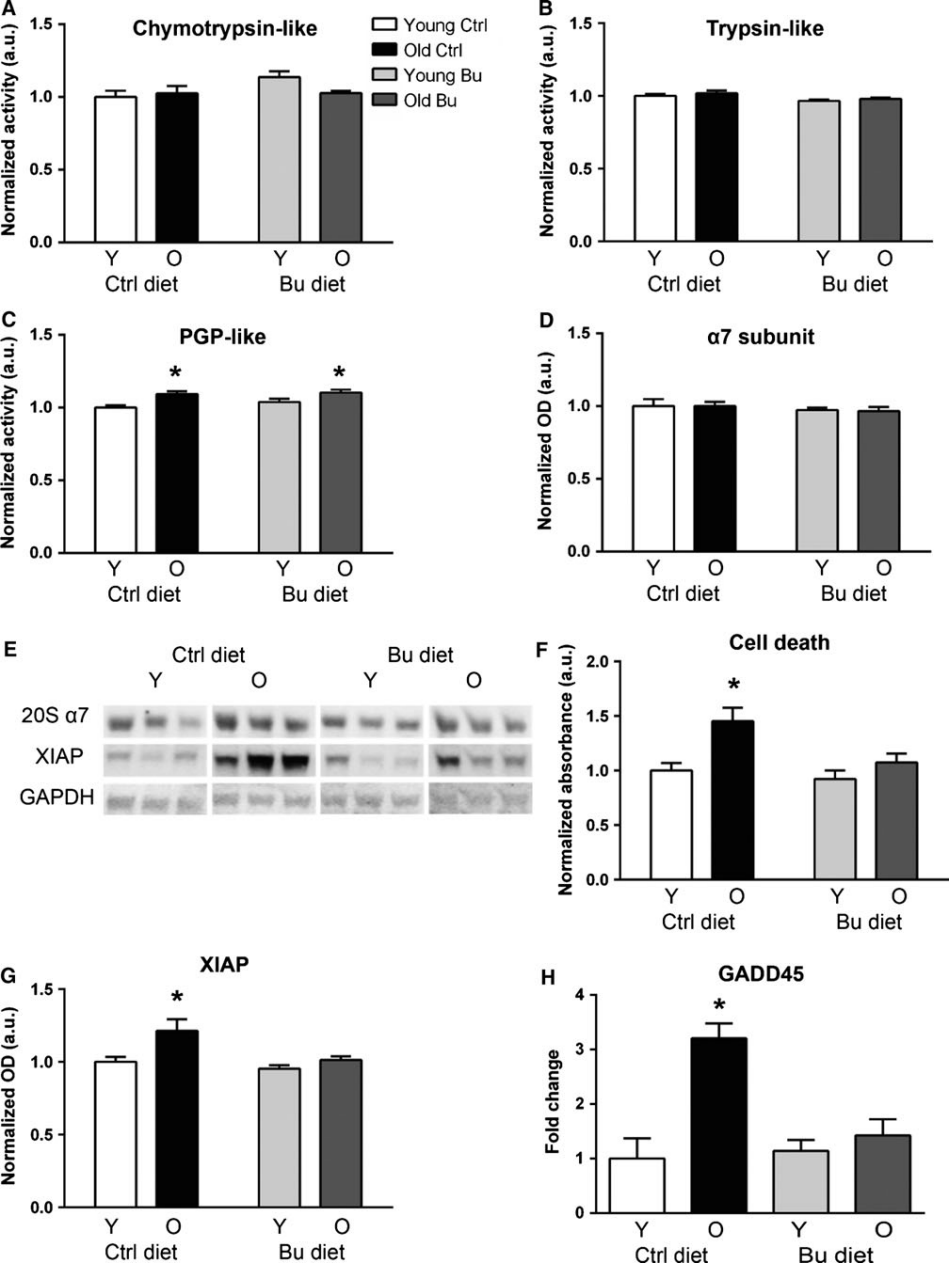
Effect of butyrate and aging on proteasome activity in skeletal muscle. Proteasome‐specific (A) chymotrypsin‐like, (B) trypsin‐like, and (C) peptidylglutamyl peptide hydrolyzing activities were determined using fluorescent substrates (*n* = 5–6). (D) Protein levels of 20S α7 subunit were determined by Western blot. (E) Western blot quantified in (D, G). (F) Results of a cell death ELISA in young and old animals treated with butyrate. (G) Protein levels of XIAP were determined by Western blot. (H) mRNA levels of Gadd45 (*n* = 8). *P < 0.05 vs. diet‐matched young determined by ANOVA with Tukey's post‐test; control young – white, control aged – black, butyrate young – light gray, butyrate aged – dark gray. All data are displayed as means with standard error.

We confirmed that butyrate increased SDH by measuring SDH activity in gastrocnemius–plantaris–soleus sections. SDH activity is used to detect oxidative fibers in skeletal muscle and is indicative of mitochondrial proliferation. We found that muscle from old mice had reduced and more dispersed staining (Fig. 3H) compared with young mice. On the other hand, SHD activity was increased in old mice fed butyrate compared with old control mice. SDH is Complex II of the mitochondrial electron transport chain in addition to being part of the citric acid cycle. Therefore, we determined the effect of age and butyrate on mitochondrial cytochrome c oxidase (COX) activity. As shown in Fig. 5, COX activity was similarly reduced in skeletal muscle from old mice fed a control diet, while old mice fed a butyrate diet maintained COX activity.

### Neither aging nor butyrate affects HDAC4‐regulated gene transcription

Loss of muscle mass during aging has been shown by our laboratory and others to involve significant denervation of muscle fibers (Valdez *et al*., 2010; Jang *et al*., 2012), and HDAC4 is elevated in skeletal muscle in response to denervation (Moresi *et al*., 2010). Consistent with these observations, we report for the first time an increase in HDAC4 levels in both control and butyrate‐treated old animals (Fig. 4A,B). HDAC4 is exported from the nucleus in response to phosphorylation, but we found no detectable phospho‐HDAC4 in young or old muscle (not shown). HDAC4 is known to regulate myogenin, a transcription factor involved in activating the muscle atrophy machinery. Thus, we determined the effect of aging and butyrate on the mRNA levels of *HDAC4*,* myogenin,* and its downstream effectors *atrogin‐1* and *MuRF1*, which are muscle‐specific E3 ligases involved in ubiquitin‐mediated protein degradation. Neither aging nor butyrate affected any of these parameters (Fig. 4C). Muscle ubiquitin ligase of the SCF complex in atrophy‐1 (Musa1), which is regulated by bone morphogenetic protein instead of myogenin (Sartori *et al*., 2013), was elevated in muscle from old mice on a control diet but not in old mice treated with butyrate. Next, we determined the expression of embryonic MHC (eMHC), a marker for regeneration that increases in muscle during aging (Edström & Ulfhake, 2005), and markers of denervation, including acetylcholine receptor α (AChRα), Runx1, and neural cell adhesion molecule (NCAM). AChRα and Runx1 were elevated >2‐fold in muscle from old mice on control and butyrate diets relative to muscle from diet‐matched young mice, and NCAM was not affected by age or diet. eMHC expression was increased approximately 3‐fold in muscle from old mice relative to young mice (Fig. 4D). In mice fed butyrate, no significant increase in eMHC expression was detected. Furthermore, central nuclei, a phenomenon that results from degeneration and reinnervation of a muscle fiber (Fig. 4E,F), were significantly elevated in mice fed a control diet, and butyrate partially prevented the age‐associated increase in central nuclei.

### Butyrate modulates markers of apoptosis but not proteasome during aging

Degradative processes, including proteasomal degradation, apoptosis, and necrosis, are associated with muscle atrophy, so we tested the effect of aging and butyrate on proteasome activity and apoptotic markers in skeletal muscle. We found no effect of aging or butyrate on chymotrypsin‐like or trypsin‐like proteasome‐specific activity and a small but significant age‐related increase in PGPH activity in aged animals fed control and butyrate diets (Fig. 5A–C). Furthermore, we found no changes due to aging or butyrate in the 20S α7 subunit of the proteasome (Fig. 5D) determined by Western blot (Fig. 5E).

Next, we determined the effect of aging and butyrate on cell death as measured by DNA fragmentation. Consistent with previous results (Dirks & Leeuwenburgh, 2004), we found elevated cell death in skeletal muscle from old animals fed a control diet (Fig. 5F). No increase was found in old animals treated with butyrate. We also found an increase in the apoptotic protein XIAP in old animals fed control diet, while no increase was found in butyrate‐treated animals (Fig. 5G). Next, we determined the expression Gadd45, a DNA damage response gene that was previously reported to be increased in skeletal muscle during aging (Edwards *et al*., 2007). Gadd45 was increased approximately 3‐fold in muscle from old mice relative to young mice (Fig. 5H). In mice fed butyrate, no significant increase in Gadd45 expression was detected.

### Butyrate modulates antioxidant activity and prevents oxidative damage in skeletal muscle

**Figure 6:**
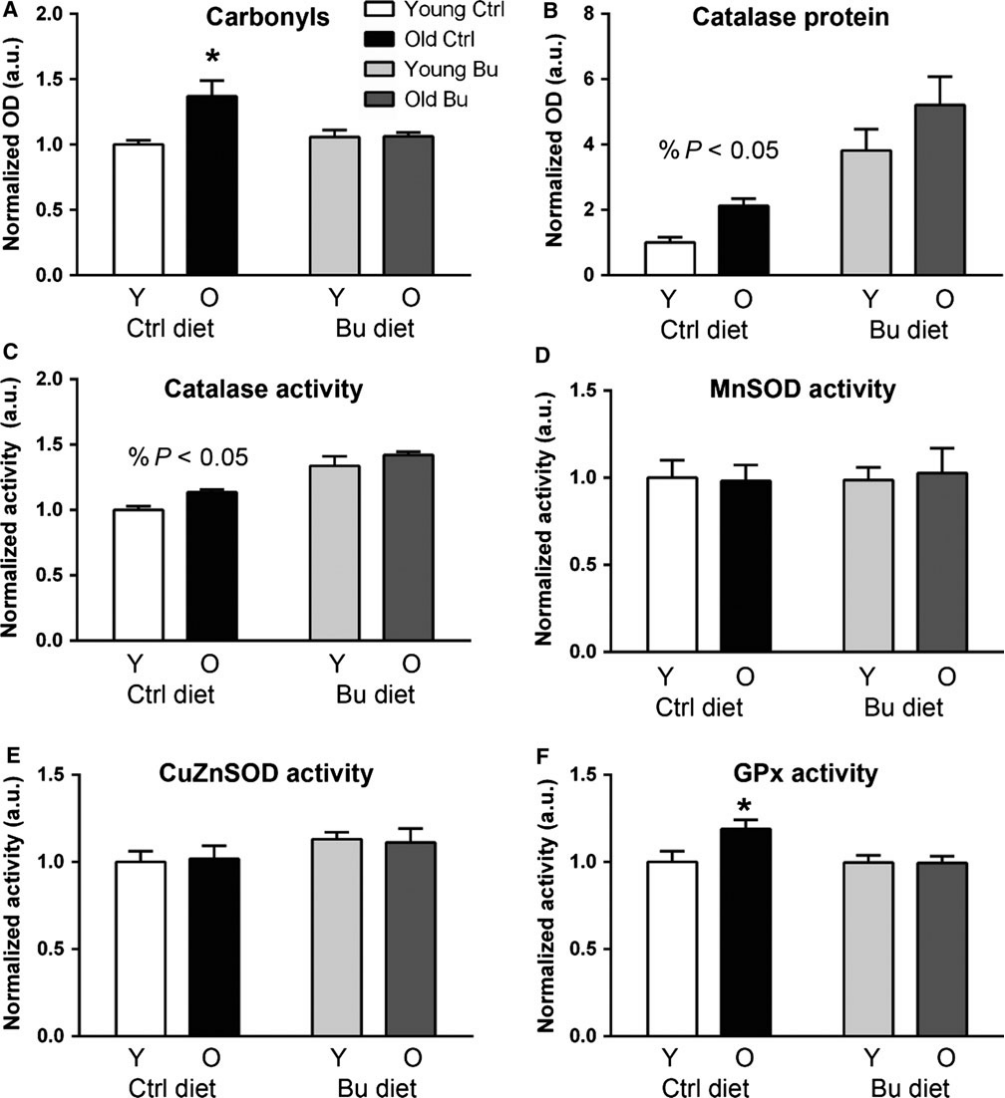
Butyrate modulates antioxidant enzyme activity in skeletal muscle during aging. (A) Protein carbonyls in young and old mice fed control butyrate diets. Catalase (B) protein and (C) activity were determined in muscle from young and old animals treated with control or butyrate diets. (D) MnSOD, (E) CuZnSOD, and (F) GPx activities were determined in young and old mice fed control and butyrate diets (*P < 0.05 vs. diet‐matched young; ^%^ *P* < 0.05 significant effect of diet; control young – white; control aged – black; butyrate young – light gray; butyrate aged – dark gray). All data are displayed as means with standard error.

Oxidative stress is observed in skeletal muscle during aging and has been implicated in age‐related muscle atrophy. Thus, we measured oxidative damage in skeletal muscle from young and old mice treated with butyrate. Protein carbonyls increased by 36% in skeletal muscle during aging. In contrast, old mice treated with butyrate had no increase in carbonyls in skeletal muscle (Fig. 6A).

Because butyrate and its derivatives have been shown to protect against oxidative stress by modulating antioxidant enzyme activity (Shimazu *et al*., 2013), we tested the effect of aging and butyrate on antioxidant and detoxification enzyme and stress–response protein levels using quantitative mass spectrometry. A significant effect of age was found for eight antioxidant enzymes (Table 2), and a significant effect of diet was observed for two enzymes: catalase and glutathione S‐transferase P. An interaction between age and diet was found for catalase, so we sought to clarify the effect of butyrate and age on catalase.

**Table 2:** Effect of age and butyrate on antioxidant enzymes

	Antioxidant, detoxification, and stress response (pmol per 100 μg total protein)				
	Young Ctrl	Old Ctrl	Young Bu	Old Bu	Significance
Akr1b1	1.1 ± 0.081	0.73 ± 0.268	1.1 ± 0.218	1.1 ± 0.055	
Aldh2	0.14 ± 0.011	0.12 ± 0.029	0.15 ± 0.024	0.19 ± 0.022	
Cat	0.06 ± 0.002	0.07 ± 0.011	0.06 ± 0.008	0.11 ± 0.013	*, ^
Gpx1	0.03 ± 0.002	0.03 ± 0.005	0.02 ± 0.004	0.03 ± 0.003	
Gsr	0.04 ± 0.004	0.06 ± 0.009	0.04 ± 0.006	0.05 ± 0.007	*
Gsta3	0.03 ± 0.001	0.02 ± 0.004	0.03 ± 0.006	0.03 ± 0.002	
Gstm1	1.0 ± 0.05	0.96 ± 0.07	0.98 ± 0.11	1.1 ± 0.08	
Gstp1	0.61 ± 0.056	0.40 ± 0.095	0.67 ± 0.107	0.71 ± 0.033	&
Hsp90b1	0.05 ± 0.006	0.05 ± 0.011	0.05 ± 0.010	0.05 ± 0.009	
Hspa1a	0.30 ± 0.025	0.34 ± 0.019	0.28 ± 0.018	0.32 ± 0.013	
Hspa5	0.27 ± 0.017	0.38 ± 0.062	0.27 ± 0.016	0.42 ± 0.023	*
Hspa9	0.17 ± 0.019	0.20 ± 0.045	0.22 ± 0.027	0.29 ± 0.050	
Msra	0.01 ± 0.001	0.01 ± 0.003	0.01 ± 0.003	0.01 ± 0.001	
Phb	0.07 ± 0.010	0.07 ± 0.015	0.08 ± 0.012	0.08 ± 0.014	
Phb2	0.06 ± 0.006	0.06 ± 0.016	0.07 ± 0.015	0.07 ± 0.015	
Prdx1	0.73 ± 0.054	0.80 ± 0.079	0.77 ± 0.067	0.71 ± 0.046	
Prdx2	0.19 ± 0.008	0.27 ± 0.030	0.24 ± 0.034	0.28 ± 0.030	*
Prdx3	0.30 ± 0.029	0.40 ± 0.047	0.34 ± 0.044	0.38 ± 0.059	
Prdx4	0.21 ± 0.030	0.32 ± 0.049	0.26 ± 0.043	0.27 ± 0.029	
Prdx5	0.13 ± 0.015	0.20 ± 0.035	0.15 ± 0.027	0.21 ± 0.028	*
Prdx6	0.17 ± 0.022	0.25 ± 0.045	0.21 ± 0.028	0.27 ± 0.045	
Sod1	0.41 ± 0.031	0.61 ± 0.046	0.40 ± 0.047	0.55 ± 0.063	*
Sod2	0.25 ± 0.025	0.27 ± 0.031	0.30 ± 0.045	0.30 ± 0.039	
Txn1	0.15 ± 0.013	0.23 ± 0.031	0.19 ± 0.021	0.22 ± 0.017	*
Txnrd1	0.09 ± 0.007	0.11 ± 0.009	0.09 ± 0.008	0.10 ± 0.005	*

Antioxidant enzyme levels were determined by quantitative mass spec in skeletal muscle (*n* = 6; pmol per 100 μg total protein; data shown is means with standard deviation.

*Significant effect of age; ^&^Significant effect of diet; ^^^Significant effect of age*diet interaction).

Catalase protein levels were determined by Western blot and were increased in mice treated with butyrate by ~4–5 fold compared with young control mice (Fig. 6B). Consistently, catalase activity was increased in young and old butyrate‐treated mice by 30%–40% (Fig. 6C). We also determined the effect of age and butyrate on glutathione peroxidase and superoxide dismutase activities. There was no effect of aging or butyrate on CuZnSOD or MnSOD activities (Fig. 6D,E). Glutathione peroxidase activity increased by 19% with aging in control‐fed mice but did not increase in old mice fed butyrate (Fig. 6F).

## Discussion

We report here for the first time the effects of long‐term treatment with the HDAC inhibitor butyrate on metabolism and age‐related muscle atrophy in mice. We found that butyrate leads to marked effects on metabolism, including reduced fat mass, improved glucose metabolism, and increased enzymes involved in oxidative metabolism in old mice. The fact that butyrate improved glucose tolerance but had no effect on insulin tolerance suggests that tissues other than skeletal muscle are involved in the metabolic effects of butyrate. Indeed, butyrate reduced gluconeogenesis as measured by the pyruvate tolerance test, suggesting that the liver may be a target organ of butyrate. These effects are consistent with previous observations and may be due to inhibition of HDAC3, which inhibits mitochondrial biogenesis and promotes hepatic gluconeogenesis (Gao *et al*., 2009; Sun *et al*., 2012; Galmozzi *et al*., 2013). Thus, HDAC3 may be a target of butyrate and future research is needed to determine the age‐related changes in HDAC3 activity.

We found that butyrate reduced muscle atrophy, prevented intramuscular fat accumulation, and elevated oxidative metabolism during aging. Because mitochondrial biogenesis has previously been associated with preservation of muscle mass and beneficial effects on metabolism (Wenz *et al*., 2009), we measured the levels of mitochondria‐related proteins. Consistent with increased SDH and COX activity in muscle from old animals treated with butyrate, we found elevated levels of TFAM and porin in response to butyrate, although neither age nor diet affected PGC‐1α. Together, these data support a role for oxidative metabolism in the reduced loss of muscle mass in butyrate‐treated mice during aging.

Signs of denervation appear as early as 18 months of age in C57Bl/6 mice (Valdez *et al*., 2010), although another study reported markers of denervation at 28 but not 22 months of age, even though atrophy was present at 22 months of age (Sousa‐Victor *et al*., 2014). Thus, we next asked whether markers of denervation were altered in the 26‐month‐old mice from this study. eMHC, a marker of newly formed fibers, has been shown to increase in response to loss of innervation (Edström & Ulfhake, 2005). The lack of eMHC expression in old muscle treated with butyrate suggests that butyrate prevents denervation or that butyrate prevents the formation of new fibers. Furthermore, butyrate prevented the age‐related increase in Gadd45a expression, a gene that mediates denervation‐induced muscle trophy that is directly regulated by HDAC4 (Bongers *et al*., 2013), and central nuclei in muscle fibers, suggesting butyrate prevents degeneration of muscle fibers. Conversely, HDAC4, AChRα, and Runx1 were increased in both control and butyrate‐treated groups, while NCAM was not affected by age or diet. Generally, these data suggest that denervation occurs in both old groups. Future studies will determine the effect of butyrate on peripheral nerves and the neuromuscular junction during aging, as one study found that HDAC inhibitors prevent motor neuron death and axonal degradation in a mouse model of ALS (Yoo & Ko, 2011).

While both proteasome activity and the E3 ligases that mark proteins for degradation are upregulated during muscle atrophy, data on the ubiquitin proteasome system and age‐related muscle atrophy vary widely; increased, decreased, and no change in proteasome activity have been reported in aged rodents (Radák *et al*., 2002; Husom *et al*., 2004; Wenz *et al*., 2009). We found no change in proteasome activity or myogenin‐regulated E3 ligases in old mice compared with young. However, we did find an increase in the bone morphogenetic protein‐regulated Musa1 in old mice fed a control, but not butyrate, diet. Further experiments are needed to determine whether bone morphogenetic protein signaling is altered by aging and butyrate. While butyrate had no effect on proteasome activity, it did alter markers of apoptosis, including preventing expression of XIAP, an effect seen with dietary restriction (Dirks & Leeuwenburgh, 2004). One other mechanism important for the maintenance of muscle mass is protein synthesis. Reduced protein synthesis has been reported in middle‐aged humans (Balagopal *et al*., 1997), although no further decline was seen at advanced ages. The effect of age and butyrate on protein synthesis needs to be determined in C57Bl/6 mice. It is possible that different mechanisms contribute to muscle loss depending on the age. Thus, a detailed time course analysis of catabolic and anabolic processes would be valuable for the field and could pinpoint potential targets for intervention.

Histone deacetylases offer a unique opportunity to determine the cause of age‐related muscle loss. Mice lacking the class II HDACs, HDAC4 and HDAC5, are protected from denervation‐induced muscle atrophy (Moresi *et al*., 2010), and we report an increase in HDAC4 during aging in skeletal muscle. These models will provide important insight into the mechanisms of age‐related muscle loss. Additionally, reduced HDAC3 is associated with mitochondrial biogenesis, and HDAC3‐specific deletion in skeletal muscle or class I‐specific inhibitors could be used to understand the role of mitochondrial biogenesis in age‐related muscle atrophy and metabolic disease. And lastly, inhibition of HDAC1 prevents muscle atrophy from disuse (Beharry *et al*., 2014). If sarcopenia results from lack of exercise and movement during aging, inhibition of HDAC1 will prevent muscle atrophy. Both genetic and pharmacological studies are crucial to clarify the role of HDACs in skeletal muscle during aging.

Oxidative stress has been proposed to play an important role in the initiation and progression of muscle atrophy during aging (Muller *et al*., 2006; Jang *et al*., 2010). Previous studies have reported elevated antioxidant enzyme activity from treatment with butyrate and HDAC inhibitors (Kang *et al*., 2002; Shimazu *et al*., 2013). Consistent with this, we found that butyrate prevented oxidative damage to proteins during aging and increased catalase activity in muscle. Contrary to the hypothesis that reduced antioxidant enzyme activity contributes to oxidative stress during aging, we found an age‐related increase in a number of antioxidants during aging, including enzymes involved in the glutathione and thioredoxin systems as well as peroxiredoxins. Dietary restriction preserves muscle mass during aging, a fact attributed to reduced oxidative stress and improved metabolism. Interestingly, dietary restriction increases the ketone body β‐hydroxybutyrate (Shimazu *et al*., 2013), a structural and functional analog of butyrate that protects against oxidative stress by increasing antioxidant enzyme activity. These data suggest a potential role for modulation of oxidative stress in age‐related muscle atrophy and the beneficial effects of butyrate on skeletal muscle.

Our data suggest that histone deacetylases contribute to muscle loss and metabolic alterations during aging and support the hypothesis that epigenetic changes contribute to aging phenotypes. The specific targets of histone deacetylases that are altered in skeletal muscle during aging are not known. In some tissues, there are few changes in the total proteome during aging (Walther & Mann, 2010; Dai *et al*., 2014). Thus, post‐translational modifications are one of the major modes of regulation that might contribute to reduced function during aging. Little is known about how histone modifications and other epigenetic alterations are changed during aging in skeletal muscle. How histone acetylation and other modifications change globally and locally in the genome and whether other epigenetic alterations occur during aging in skeletal muscle are promising research directions.

Finally, we cannot rule out the possibility that butyrate exerts its effects independent of the inhibition of histone deacetylases. Other than HDAC inhibition, butyrate has two effects in the cell; it is a fatty acid that can be used as an energy source, and it can bind G‐protein‐coupled receptors that are involved in the resolution of inflammation in the gut (Maslowski *et al*., 2009). Whether these receptors have a function in skeletal muscle needs to be determined. Our data suggest butyrate and potentially other HDAC inhibitors could be used to treat age‐related metabolic disease and sarcopenia.

## Experimental procedures

### Mice and diet

We initiated this study in 6‐month‐old (young) and 16‐month‐old (old) female C57Bl/6J mice. Mice were multiply housed (2–5/cage) in specific pathogen‐free conditions and fed diets containing 5% sodium butyrate (butyrate) or a control diet containing an equivalent amount of sodium in the form of sodium chloride (Teklad animal diets TD.10434 and TD.110009, Harlan Laboratories). In this formulation, butyrate was previously shown to inhibit HDAC activity (Gao *et al*., 2009) and increase histone acetylation (Walsh *et al*., 2015) in skeletal muscle. Young and old cohorts were fed control or butyrate diets for 8–10 months. All *in vivo* procedures were performed in young and old groups at 12 and 24 months of age, respectively, and all old mice were sacrificed at 26 months of age. Mice were sacrificed by carbon dioxide inhalation followed by cervical dislocation and tissues were dissected, weighed, and flash‐frozen in liquid nitrogen for further analysis. All procedures were approved by the Institutional Animal Care and Use Committees of the University of Texas Health Science Center and the Audie L. Murphy Memorial Veterans Hospital (San Antonio, TX, USA).

### Protein preparation and Western blot

Frozen muscle was homogenized in buffer containing 50 mM Tris‐HCl buffer with 150 mM NaCl, 1% Nonidet P‐40, 0.25% sodium deoxycholate and protease inhibitor cocktail. Protein concentration was determined by the Bradford method. SDS‐PAGE and Western blotting were performed as previously described (Shi *et al*., 2013). Primary antibodies are detailed in Supplemental Experimental Procedures. Bands were imaged using chemiluminescent substrate (Thermo Scientific, Waltham, MA, USA) and a Typhoon imager (GE Healthcare Life Sciences Pittsburgh, PA, USA).

### Carbonyl assay

Carbonyls were determined as previously described (Hamilton *et al*., 2013).

### Mass spectrometry

Quantitative mass spectrometry was performed as previously described (Rindler *et al*., 2012) and detailed in Supplemental Experimental Procedures.

### Quantitative magnetic resonance imaging

Percentage fat and lean mass were measured using the EchoMRI (Echo Medical Systems, Houston, TX, USA) qMRI system.

### Whole‐body oxygen consumption

Oxygen consumption and carbon dioxide production were measured for 24 h using a MARS indirect calorimetry system (Sable Systems International, Las Vegas, NV, USA) as previously described (Shi *et al*., 2013).

### Spontaneous activity

Animals were housed individually in Plexiglas cages surrounded by a 2.5‐cm grid of infrared sensors in the *x*,* y*, and *z* planes that record movement throughout a 48‐h monitoring period. Data from the final 40 h were used for analysis.

### Cell death ELISA

Cell death ELISA (Roche Diagnostics Corp., Indianapolis, IN, USA) was performed according to the manufacturer's directions. This assay measures the DNA fragmentation that is characteristic of apoptotic cell death by quantifying cytosolic mono‐ and oligonucleosomes (Dirks & Leeuwenburgh, 2004).

### Glucose, pyruvate, and insulin tolerance tests

Mice were fasted for 16 h for glucose and pyruvate tolerance tests and 6 h for insulin tolerance tests. Glucose in saline was injected intraperitoneally for GTT and PTT (1.5 mg glucose per kg body weight and 1.5 mg glucose per kg body weight, respectively), and recombinant human insulin (Humulin; Eli Lilly, Indianapolis, IN, USA) was injected intraperitoneally (1 U kg^−1^) for ITT. Blood glucose was monitored at indicated time points using a One‐Touch Ultra glucometer.

### mRNA isolation and reverse transcription

RNA isolation and reverse transcription were performed as previously described (Walsh *et al*., 2015).

### Real‐time polymerase chain reaction

Quantitative PCR was performed using Power SYBR Green (Applied Biosystems, Foster City, CA, USA) and primers as previously described (Walsh *et al*., 2015) and detailed in Supplemental Experimental Procedures.

### Histology

Gastrocnemius–plantaris with soleus was rapidly frozen in isopentane cooled by liquid nitrogen. Muscle was sectioned into six‐micron sections using a cryostat (Bright Instruments Co., Huntingdon, UK) and used staining protocols described in the Supplemental Experimental Procedures. All imaging was performed with a Nikon TE‐2000 microscope (Nikon, Inc., Melville, NY, USA).

### MHC composition

MHC composition was determined by SDS‐PAGE as previously described (Qaisar *et al*., 2012).

### Statistics

Student's *t*‐test was used for two group, non‐repeated measures. ANOVA with repeated measures and two‐way ANOVA with corrections for multiple comparisons were used as appropriate with IBM SPSS statistical software (Armonk, NY, USA).

## Supplemental experimental procedures

### Acid extract of histones

Frozen tissue was homogenized in 20 mM Tris pH 7.4 with 0.1% Triton X‐100 and trichostatin A on ice. Homogenates were centrifuged for 15 min at 1000 *g* and 4 °C. Supernatants were discarded and pellets were resuspended in 0.2 m H_2_SO_4_ and incubated overnight at 4 °C on a rocker. The debris was pelleted, and acid‐soluble protein was precipitated by incubating with trichloroacetic acid for 4 h at 4 °C. Precipitated protein was pelleted and washed three times with Tris pH 7.4 buffer and then resuspended in Tris pH 7.4 with 0.1% sodium dodecyl sulfate. Protein concentration was determined by bicinchoninic acid assay (Pierce, Rockford, IL, USA) using manufacturer's recommended protocol.

### Antioxidant enzyme activity

Frozen skeletal muscle was homogenized in 20 mM Tris pH 7.5 with 0.05% Triton X and protease inhibitors, freeze–thawed three times and centrifuged at 4 °C for 10 min at 14 000 *g*. Protein content in the supernatant was quantified using Bradford reagent (Bio‐Rad Laboratories, Hercules, CA, USA) and used for the following assays.

### Superoxide dismutase

SOD activity was measured with non‐reducing PAGE as previously described (Shi *et al*., 2013).

### Catalase

Catalase activity was measured in 96‐well plates using an Amplex Red/horseradish peroxidase method. Homogenates were added to 40 μm H_2_O_2_ for 30 min at 37 °C. A total of 35 μm Amplex Red and 0.5 U HRP were added to react with remaining H_2_O_2_, and fluorescence was read with a Fluoroskan Ascent type 374 multiwell plate reader. Catalase activity is thus inversely proportional to the amount of fluorescent product, resorufin.

### Glutathione peroxidase

Glutathione peroxidase activity measured with an indirect, coupled assay with glutathione reductase, glutathione, and limiting nicotinamide adenine dinucleotide phosphate. Homogenates were added to a buffer containing 5 mm nicotinamide adenine dinucleotide phosphate, 42 mm GSH, 10 U mL^−1^ glutathione reductase, and 30 μm cumene hydroperoxide. Decomposition of nicotinamide adenine dinucleotide phosphate was monitored at 340 nm using a spectrophotometer (Beckman Coulter DU 800 Chaska, MN, USA).

### Quantitative PCR analysis

Fold change was determined with the ΔΔ*C*t method. Technical triplicates were averaged, and the Δ*C*t values were calculated by subtracting the β‐actin *C*t value. All groups were normalized to the young control group, and fold change was determined by calculating 2^−ΔΔ*C*t^.

### Proteasome activity

Chymotrypsin‐like protease activity was measured using fluorescent substrates for chymotrypsin [Succinyl‐Leu‐Leu‐Val‐Tyr‐(7‐amino‐4‐methylcoumarin) (Suc‐LLVY‐AMC)], trypsin (Boc‐Leu‐Leu‐Arg‐AMC), and peptidylglutamyl peptide hydrolyzing (Z‐Leu‐Leu‐Glu‐AMC) activities, which were acquired from Ezno Life Sciences (Farmingdale, NY, USA): Frozen gastrocnemius–plantaris was homogenized in a buffer containing 50 mm HEPES, 20 mm KCl, 5 mm MgCl2, 1 mm dithiothreitol, and 2 mm ATP, pH 7.5. This homogenate was centrifuged for 30 min at 10 000 *g* at 4 °C. The supernatant was removed, and protein concentration was determined by the Bradford method. Protein was incubated with 100 μm substrate for 60 min at 37 °C. Fluorescence was measured using a Fluoroskan Ascent type 374 multiwell plate reader (excitation 355 nm, emission 460 nm).

### Hematoxylin and treosin staining

Muscle sections were stained with Gill hematoxylin and counterstained with treosin. Cross‐sectional area and minimum Feret's area were determined from five images per mouse (>750 fibers per group) with imagej software (NIH, Bethesda, MD, USA).

### Oil Red O staining

Muscle sections were air‐dried, fixed in 4% paraformaldehyde, and stained in 0.5% Oil Red O for 15 min. Afterward, sections were rinsed and dipped in Gill hematoxylin for 10 s.

### Succinate dehydrogenase and cytochrome c oxidase activity

Succinate dehydrogenase and cytochrome c oxidase activity was determined as previously described (Lustgarten *et al*., 2011).

### Nerve conduction study

Nerve conduction velocity was determined as previously described (Walsh *et al*., 2014). Briefly, proximal ankle electrodes were stimulated, and the response was recorded at the distal foot electrodes placed dorsally over all five digits. The latency and distance between electrodes were measured, and then the stimulating electrodes were placed at the sciatic notch. The nerve was stimulated again, and the resultant latency was subtracted from the initial ankle‐foot latency. This difference was divided between the distance between the notch and ankle to determine velocity.

### Bone mineral density and bone mineral content

Bone mineral density and bone mineral content of collected bones at sacrifice were measured by dual energy x‐ray absorptiometry using a Lunar PIXImus mouse bone densitometer (General Electric , Fitchburg, WI, USA), and data analysis was carried out manually with piximus software as previously described (Rahman *et al*., 2011). Calibration of the instrument was conducted as suggested by the manufacturer. An aluminum/lucite phantom was placed on the specimen tray and measured 25 times without repositioning. Thereafter, the phantom was analyzed daily before animal testing for quality control purposes. During the measurements, the bone samples were placed on specimen tray. Upon completion of the scanning, bone mineral density and bone mineral content were determined in the following bone areas using the piximus software.

### Passive avoidance

On day 1, mice were placed in a lit chamber in the GEMINI testing device (San Diego Instruments; San Diego, CA, USA), and 30 s later a door opened to an unlit but otherwise identical chamber. Mice were removed after entering the unlit chamber. On day 2, an identical setup was used except that upon entering the unlit chamber, a 2‐s foot shock (2 mA) was delivered through the grid floor. Mice were left in the unlit chamber for an additional 30 s. On day 3, mice were placed in the lit chamber, and the door to the unlit chamber opened after 30 s. The amount of time it took for the mouse to cross from the lit chamber to the unlit chamber was recorded, with a maximum time of 300 s. Mice that did not enter the unlit chamber on day 1 or 2 were not subject to further testing.

### Mass spectrometry

Muscle samples were homogenized in 10 mm Tris, pH 7.8, 150 mm NaCl, 1% Triton‐X with protease inhibitor cocktail (Calbiochem Set III, EDTA‐free; EMD Millipore; Billerica, MA, USA). Protein concentration was determined and a volume equivalent to 100 μg of total protein taken for analysis. Bovine serum albumin was added to samples to serve as an internal standard. The samples were incubated at 80 °C for 15 min in 10% SDS. Proteins were precipitated with 80% acetone at −20 °C overnight. The protein pellet was dissolved and separated using SDS‐PAGE. The gel was fixed and stained with GelCode Blue, and the 1.5‐cm lane was cut out of the gel. Gel pieces were washed, reduced with DTT, alkylated with iodoacetamide, and digested with trypsin at room temperature overnight. Digested peptides were extracted with 50% methanol and 10% formic acid. This extract was dehydrated and dissolved in 1% acetic acid.

Samples were analyzed using selected reaction monitoring with a triple quadrupole mass spectrometer (ThermoScientific TSQ Vantage, Waltham, MA, USA) with a splitless capillary column HPLC system (Eksigent, Dublin, CA, USA). Samples were injected into a C18 column (Phenomenex, Jupiter C18) and eluted with a gradient of acetonitrile in 0.1% formic acid. Pinpoint (ThermoScientific) was used to monitor and process data from each peptide. Two housekeeping proteins, voltage‐dependent anion‐selective channel protein 1 and heat‐shock protein 1, and bovine serum albumin were used to normalize peptides representing glycolytic, oxidative, and antioxidant enzyme proteins using the best flyer approach. Several proteins were not detected, including Pdk1 and Pdk4 in the oxidative panel, AldoB, Pfk,l and Pygb in the glycolytic panel, and Gpx4 and Nnt in the antioxidant panel. False discovery rate was determined with Benjamini–Hochberg test with α = 0.05.

### Primers for quantitative polymerase chain reaction

Actin (f‐CTGTCCACCTTCCAGCAGATGT; r–CGCAACTAAGTCATAGTCCGCC)

HDAC4 (f–CTTGAGCTGCTGCAGCTTC; r–CAGATGGACTTTCTGGCCG)

myogenin (f–ACTCCCTTACGTCCATCGTG; r–CAGGACAGCCCACTTAAA)

atrogin‐1 (f–CTCTGTACCATGCCGTTCCT; r–GGCTGCTGAACAGATTCTCC)

MuRF1 (f–ACCTGCTGGTGGAAAACATC; r–AGGAGCAAGTAGGCACCTCA)

Gadd45a (f–GCTGCCAAGCTGCTCAAC; r–TCGTCGTCTTCGTCAGCA)

TFAM (f–GAGCTTGTAAATGAGGCTTG; r–CACTTCGACGGATGAGATC)

PGC‐1α (f–AGCCGTGACCACTGACAACGAG; r–GCTGCATGGTTCTGAGTGCTAAG)

eMHC (f–GACTCAGCCGACACCATGAG; r–TTTTCCGACTTGCGGAGGAA)

### Primary antibodies used for Western blotting

HDAC4 (H‐92), phospho‐HDAC4 (Ser 632), tubulin, and mitochondrial transcription factor A (TFAM) antibodies were obtained from Santa Cruz Biotechnology, Dallas, TX USA; porin antibody was obtained from Abcam, Inc., Cambridge, MA, USA; GAPDH antibody was obtained from Cell Signaling Technology, Danvers, MA, USA; X‐linked inhibitor of apoptosis protein (XIAP) was obtained from MBL International, Woburn, MA, USA; 20S α7 subunit antibody was obtained from Enzo Life Sciences, Farmingdale, NY, USA.

## Author contributions

MEW planned and performed experiments, interpreted data, and wrote the manuscript. AB performed experiments and edited the manuscript. LS, KS, QR, MMR, and MK performed experiments. HVR planned experiments, interpreted data, and edited the manuscript.

## Funding info

This work was supported by P01 020591 (HVR) and an NIA Training Grant on the Biology of Aging (MEW, T32AG021890).

## Conflict of interest

None declared.

## Supporting information


**Fig. S1.** Body temperature, food consumption, body weight, and tissue weight in old mice.

**Fig. S2.** Effect of age and butyrate on functional parameters.
