# Supplementary figures and images for: The histone deacetylase inhibitor butyrate improves metabolism and reduces muscle atrophy during aging
Source: Aging Cell. 2015 Aug 20;14(6):957–70. doi: 10.1111/acel.12387 (PMC4693467; doi:10.1111/acel.12387)

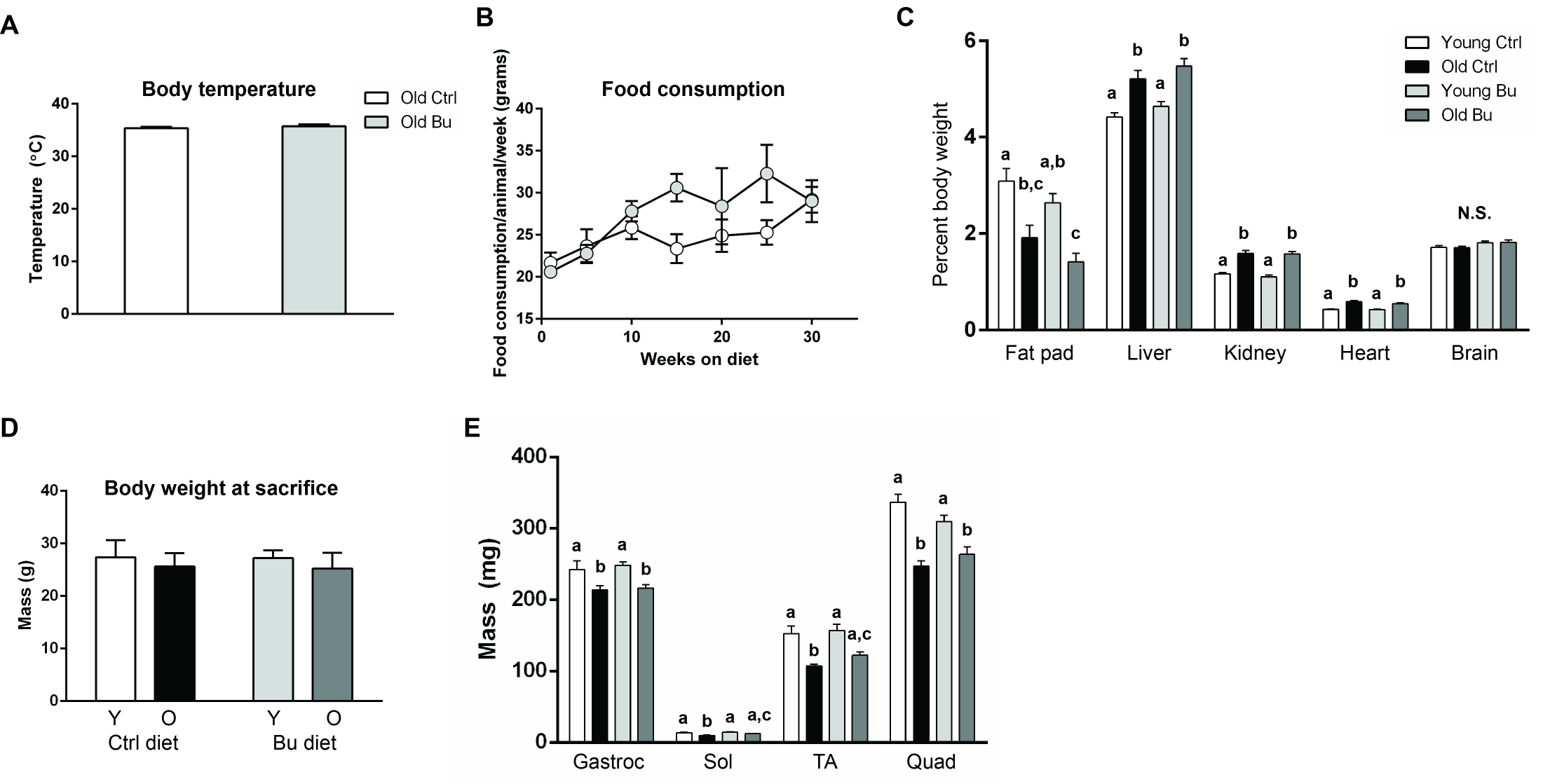

Supplement: Supplementary file 1 — Fig. S1. Body temperature, food consumption, body weight, and tissue weight in old mice. [file ACEL-14-0957-s001.tif]

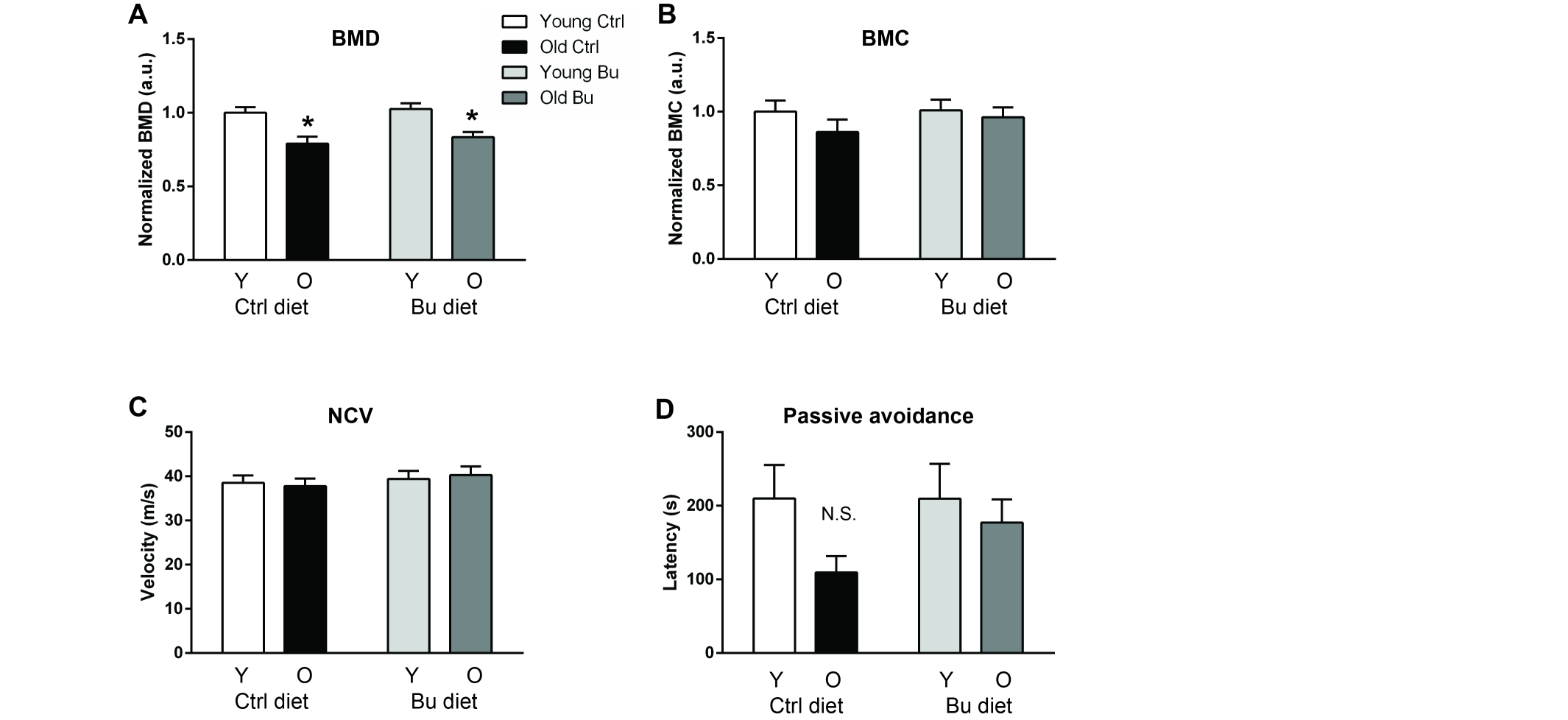

Supplement: Supplementary file 2 — Fig. S2. Effect of age and butyrate on functional parameters. [file ACEL-14-0957-s002.tif]
